# Analysis of fracture resistance in teeth obturated with graphene oxide-modified sealers

**DOI:** 10.6026/973206300210759

**Published:** 2025-04-30

**Authors:** Unmesh Khanvilkar, Arpita Anand, Anshu Nishchhal, Jyoti Kumari, Peeyush Kumar Chaudhary, Vinay Rao

**Affiliations:** 1 Department of Micro Dentistry, MUHS, Mumbai Regional Center, Mumbai, Maharashtra, India; 2 Department of Conservative Dentistry and Endodontics, Dental College Azamgarh, Azamgarh, Uttar Pradesh, India; 3 Department of Conservative Dentistry, Endodontics and Aesthetic Dentistry, AMC Dental College, Ahmedabad, Gujarat, India

**Keywords:** Fracture resistance, graphene oxide, root canal sealer, obturation, endodontics, biomechanical properties

## Abstract

Analysis of tooth resistance after obtaining them with regular sealers in addition to calcium silicate based ones and graphene oxide modified versions is of interest. Hence, 3 groups containing sixty premolars underwent universal testing machine evaluation. The mean fracture resistance value for teeth treated with graphene oxide (GO)-modified sealer reached 530 ± 22 N in Group 3 which resulted in significantly better results than the other tested groups (p < 0.05). We show that GO-added sealers lead to superior structural reinforcement for teeth which have undergone root canal treatment. Introducing GO into endodontic treatment regimens would lower the risk of fractures and result in better treatment performance.

## Background:

Root canal treatment is a widely performed procedure aimed at eliminating infection and preserving the function of diseased teeth. However, endodontically treated teeth (ETT) often exhibit compromised mechanical properties and reduced fracture resistance due to the cumulative effects of caries, endodontic access cavity preparation and chemical alterations in dentin following exposure to irrigants and medicaments [1]. MgO significantly improved the anti-biofilm ability and ZrO2 enhanced the radiopacity of the premixed sealer [2]. The loss of structural integrity significantly increases the risk of vertical root fractures, which is a major cause of endodontic failure and tooth loss [3]. In this context, the choice of root canal sealer plays a critical role not only in achieving a hermetic seal but also in reinforcing the root structure to enhance its resistance to fracture [4]. Graphene oxide (GO), a nanomaterial with unique physicochemical properties, has gained attention in endodontics due to its exceptional mechanical strength, antimicrobial efficacy and biocompatibility [5].

Tricalcium silicate sealer sets well with dentinal fluid, but its behavior in the body may differ from lab results [6]. It has been reported that the incorporation of GO into root canal sealers can significantly improve their mechanical properties, bonding strength and resistance to microleakage, potentially enhancing the overall fracture resistance of ETT [7, 8]. Conventional root canal sealers, such as epoxy resin-based (*e.g.*, AH Plus) and calcium silicate-based (*e.g.*, BioRoot RCS) materials, have been extensively used in endodontic therapy. However, despite their favorable sealing ability, their contribution to reinforcing the dentin structure remains a concern, as they may not sufficiently counteract the loss of dentinal stiffness and microstructural integrity [9, 10]. Several studies have demonstrated that GO-modified sealers exhibit superior mechanical performance compared to conventional sealers. GO's reinforcement effect is attributed to its high surface area, excellent dispersion and strong interfacial interaction with the sealer matrix, which enhances the adhesion between the sealer and dentinal walls [11, 12]. Additionally, GO's antimicrobial properties contribute to reducing bacterial adhesion and biofilm formation, which are critical factors in preventing reinfection and ensuring long-term success in endodontic therapy [13, 14]. Furthermore, GO's ability to increase fracture toughness and elastic modulus in composite materials suggests its potential in improving the structural resilience of obturated teeth [15, 6]. Given these advantages, this study aims to compare the fracture resistance of teeth obturated with conventional and GO-modified sealers. By evaluating the mechanical performance of GO-enhanced sealers in root-filled teeth, this research seeks to determine whether their application can provide clinically significant benefits in endodontics, particularly in reducing the risk of post-treatment fractures and enhancing the longevity of endodontically treated teeth [7, 8]. Therefore, it is of interest to analyse fracture resistance in teeth obturated with graphene oxide-modified sealers.

## Materials and Methods:

## Sample selection:

Sixty freshly extracted human premolars with single roots and mature apices were selected for this study. Teeth with cracks, fractures, resorptive defects, or previous endodontic treatment were excluded. The selected teeth were stored in 0.9% saline solution at room temperature until use.

## Root canal preparation:

All teeth were decoronated to standardize a root length of 15 mm. Root canal instrumentation was performed using a rotary nickel-titanium system up to size F4 (Protaper Universal, Dentsply Maillefer, Switzerland). The canals were irrigated with 5.25% sodium hypochlorite (NaOCl) and 17% ethylenediaminetetraacetic acid (EDTA) for smear layer removal, followed by final rinsing with distilled water. The canals were dried using sterile paper points.

## Experimental groups and obturation:

The specimens were randomly divided into three groups (n=20) based on the type of root canal sealer used:

[1] Group 1: Obturated with gutta-percha and an epoxy resin-based sealer (AH Plus, Dentsply Maillefer).

[2] Group 2: Obturated with gutta-percha and a calcium silicate-based sealer (BioRoot RCS, Septodont).

[3] Group 3: Obturated with gutta-percha and a graphene oxide (GO)-modified sealer (custom formulation).

All canals were filled using the cold lateral compaction technique. Excess gutta-percha was removed and the coronal access was sealed with a temporary filling material. The specimens were stored at 37°C in 100% humidity for one week to allow sealer setting.

## Fracture resistance testing:

Each specimen was embedded in acrylic resin blocks, leaving 2 mm of the root exposed. The samples were subjected to a vertical compressive force using a universal testing machine (Instron, USA) at a crosshead speed of 1 mm/min until fracture occurred. The maximum load at fracture was recorded in Newtons (N).

## Statistical analysis:

The collected data were analyzed using one-way analysis of variance (ANOVA) to compare fracture resistance among the groups. Post-hoc Tukey's test was applied for pairwise comparisons. A significance level of p < 0.05 was set. All statistical analyses were performed using SPSS software (version 25.0, IBM Corp, USA).

## Results:

The fracture resistance values of the three experimental groups were recorded and analyzed. The mean fracture resistance values, along with their respective standard deviations, are presented in Table 1. Table 1 shows that the highest mean fracture resistance was observed in Group 3 (Graphene Oxide-Modified Sealer) with 530 ± 22 N, followed by Group 2 (Calcium Silicate-Based Sealer) with 480 ± 25 N and the lowest in Group 1 (Epoxy Resin-Based Sealer) with 450 ± 20 N. Statistical analysis using one-way ANOVA indicated a significant difference in fracture resistance among the groups (p < 0.05). Pairwise comparisons using Tukey's post-hoc test revealed that Group 3 had significantly higher fracture resistance compared to Groups 1 and 2 (p < 0.05), while Group 2 showed a non-significant but higher mean value than Group 1. These findings suggest that incorporating graphene oxide into the root canal sealer enhances the fracture resistance of endodontically treated teeth compared to conventional sealers.

## Discussion:

The fracture resistance of endodontically treated teeth (ETT) is a critical factor influencing their longevity and success. Loss of coronal and radicular dentin during cavity preparation and instrumentation weakens the tooth structure, making it more susceptible to fracture [1]. This study assessed the impact of graphene oxide (GO)-modified sealers on the fracture resistance of obturated teeth compared to conventional epoxy resin-based and calcium silicate-based sealers. Our findings demonstrated that teeth obturated with GO-modified sealers exhibited significantly higher fracture resistance than those obturated with conventional sealers. This enhancement can be attributed to the superior mechanical properties of GO, including its high tensile strength and ability to reinforce composite materials [3, 4]. Previous studies have shown that the addition of GO to dental materials improves their mechanical durability and bonding strength, which could explain the increased fracture resistance observed in this study [5]. Calcium silicate-based sealers (Group 2) exhibited greater fracture resistance than epoxy resin-based sealers (Group 1), though the difference was not statistically significant. This finding aligns with previous research indicating that calcium silicate-based sealers promote biomineralization and dentin reinforcement, contributing to improved mechanical stability [7]. The radiopacity of root canal sealers increases when combined with gutta-percha, but the overall radiopacity of the filling depends on the sealer's type and thickness [8]. The release of calcium ions from these sealers enhances the formation of hydroxyapatite-like structures within dentinal tubules, thereby increasing fracture resistance [9]. However, their mechanical properties remain inferior to those of GO-modified sealers, which offer additional reinforcement through strong interfacial adhesion and Nano structural interactions [10]. Release high levels of Ca and P ions that could remineralize and strengthen the tooth structures [11]. Dual-cured resin sealer has recently been proposed as an innovative endodontic filling material [12]. The significant increase in fracture resistance in Group 3 (GO-modified sealers) highlights the potential of graphene-based materials in endodontics. GO has been shown to enhance the flexural strength and bonding ability of sealers, reducing microcracks and reinforcing dentinal walls. Endodontic sealers that possess both optimum flow ability and antimicrobial properties may theoretically assist in the elimination of microorganisms located in confined areas of the root canal system [13]. Chitosan paste with graphene oxide showed strong antibacterial action against E. faecalis and supported stem cell growth, making it a promising option for root canal treatment [14, 15].

## Conclusion:

We show that graphene oxide added sealers lead to superior structural reinforcement for teeth which have undergone root canal treatment. Introducing graphene oxide into endodontic treatment regimens would lower the risk of fractures and result in better treatment performance. Long-term clinical evaluations and the effect of graphene oxide modified sealers on other endodontic parameters, such as sealing ability and cytotoxicity is needed in future studies.

## Figures and Tables

**Table 1:** Fracture resistance results

**Group**	**Mean Fracture Resistance (N)**	**Standard Deviation (N)**
Epoxy Resin-Based Sealer (Group 1)	450	20
Calcium Silicate-Based Sealer (Group 2)	480	25
Graphene Oxide-Modified Sealer (Group 3)	530	22
